# Supplementary material for: Patterns of Intron Gain and Loss in Fungi
Source: PLoS Biol. 2004 Nov 30;2(12):e422. doi: 10.1371/journal.pbio.0020422 (PMC532390; doi:10.1371/journal.pbio.0020422)
Supplement: Table S1 — Also available at http://genes.mit.edu/NielsenEtAl/. (4.3 MB ZIP). [file pbio.0020422.st001.zip › NielsenEtAl/html/504.html]

AN6679.1.NCU03038.1.MG00349.1.FG07048.1


```
 CLUSTAL W (1.82) Multiple Sequence Alignments - Introns Inserted


Sequence 1: NCU03038.1	151 aa
Sequence 2: MG00349.1	151 aa
Sequence 3: FG07048.1	151 aa
Sequence 4: AN6679.1	169 aa
Alignment Length: 169 aa
Number Identitical Residues: 121 aa
Alignment Score (without introns) 4942


MG00349.1 	MGRMHSNGKGISASAIPYSRNPPAWLKTTPDQVVEQICKLARKGATPSQIGVVLRDSHGI
NCU03038.1	MGRMHSKGKGISASAIPYSRNPPSWLKTTPEQVVDQICKLAKKGATPSQIGVILRDSHGI
FG07048.1 	MGRLHSKGKGISASALPYSRSSPAWLKTTPEQVVEQISKLARKGATPSQIGVILRDSHGI
AN6679.1  	MGRLHSKGKGIASSALPYSRTPAPWVKITPEQVVDHICKLARKGASPSQIGVVLRDSHGV
          	***:**:****::**:****....*:* **:***::*.***:***:******:******:

MG00349.1 	AQVKIVTG1NKILRILKSNG1LAPDIPEDLYMLIKK0------------------AVSVR
NCU03038.1	AQVKVVTG1NKILRILKSNG1LAPDLPEDLYMLIKK0------------------AVAVR
FG07048.1 	AQVKHVTG1NRILRILKSSG1LAPELPEDLYMLIKK0------------------AVAVR
AN6679.1  	AQVKTVTG1NKILRILKSNG1LAPEIPEDLYHLIKK~VNYFGLQLFSGPANYLVQAVAVR
          	**** *** *:*******.* ***::***** ****  .  . .  :..:.   .**:**

MG00349.1 	KHLERNRKDKDSKFRLILIESRIHR~LARYYKTVGVLPPTWKYESATASTLVA
NCU03038.1	KHLERNRKDKDSKFRLILIESRIHR~LARYYKTVGVLPPTWKYESSTASTIVS
FG07048.1 	KHLERNRKDKDSKFRLILIESRIHR2LARYYKTVGVLPPTWKYESATASTIVA
AN6679.1  	KHLERNRKDKDSKFRLILIESRIHR~LSRYYKTVGVLPPTWKYESATASTMVA
          	************************* *:*****************:****:*:
```
